# Comparing MD-PhD trainee experiences by disciplinary background

**DOI:** 10.1172/jci.insight.199316

**Published:** 2026-04-07

**Authors:** Cambray Smith, Evans K. Lodge, C. Ray Cheever, Seth M. Holmes, Anna R. Kahkoska

**Affiliations:** 1 Department of Health Policy and Management,; 2 School of Medicine, and; 3 Department of Epidemiology, University of North Carolina at Chapel Hill, Chapel Hill, North Carolina, USA.; 4 Department of Family Medicine, Memorial Hospital, South Bend, Indiana, USA.; 5 Department of Nutrition, University of North Carolina at Chapel Hill, Chapel Hill, North Carolina, USA.; 6 Division of Society and Environment, University of California at Berkeley, Berkeley, California, USA.; 7 University of Barcelona, ICREA Catalan institution for Research and Advanced Study, Barcelona, Spain.

## Abstract

MD-PhD trainees increasingly pursue PhDs in social sciences, humanities, and public health (SSHPH). We characterized SSHPH trainee experiences and compared them with those of peers in traditional biomedical disciplines. From March to July 2023, a nationwide survey was sent to US MD-PhD programs that accept SSHPH trainees. Both SSHPH and non-SSHPH trainees participated in a survey focused on belonging, challenges and barriers, funding, and leadership recommendations. Quantitative data were analyzed using Fisher’s exact tests, Student’s *t* tests, and Wilcoxon’s rank-sum tests. Qualitative comments were analyzed using a hybrid deductive-inductive approach. 234 MD-PhD trainees across the US participated, with 111 (47.4%) in SSHPH and 123 (52.6%) in non-SSHPH disciplines. Overall, there were many similarities between trainees across disciplinary groups, but small and consistent differences were noted among SSHPH trainees, including decreased belonging, difficulty identifying role models, and increased work requirements during graduate school. Respondents had 5 recommendations for MD-PhD leaders and 3 recommendations for the NIH, such as integrating SSHPH scholars into speaker series and incentivizing funding parity. Limitations include high percentages of missing responses. This exploratory study provides insights into SSHPH MD-PhD trainee experiences, highlighting similarities and unique needs that can be addressed within and across MD-PhD programs.

## Abstract 2

What’s it like to pursue an MD-PhD in humanities, social sciences, and public health? A new survey provides insights from current trainees, including concrete recommendations for program directors and the NIH.

## Introduction

Physician-scholars bridge research and the practice of medicine (1, 2). MD-PhD training represents a major route through which physician-scholars develop their skill sets, with many students in the US training through federally supported Medical Scientist Training Programs (MSTPs) (3).

Within the last several decades, a growing number of MD-PhD students have pursued doctoral training in social sciences, humanities, and public health (SSHPH), both within and outside of MSTPs. Historically, most students completed degrees in traditional biomedical and basic sciences, including disciplines such as cell biology, neuroscience, and immunology. The more recent disciplinary diversification of physician-scholars parallels a growing recognition of the social and public health factors that shape medical care and research, with estimates that 80%–90% of health outcomes result from social, behavioral, economic, and environmental conditions compared with only 10%–20% from clinical care (4). These interdisciplinary perspectives can enrich training cohorts and broaden the effect of the physician-scholar workforce, even among those who do not conduct SSHPH research (5–8).

Although the proportion of MD-PhD trainees in SSHPH is growing, little empirical work has focused on trainees’ experiences. To our knowledge, the single study published about SSHPH MD-PhD experiences is a 2017 survey by Holmes et al. of alumni (mean age 55 years), assessing perspectives and outcomes related to training (9). The authors found that 90% completed residency, 98% were engaged in research, and 88% were based at academic institutions (9). Roughly three-fourths of participants were optimistic about careers combining medicine and SSHPH, with enthusiasm affected by encouraging factors (e.g., passion for work, supportive mentorship, and institutional support) and discouraging factors (e.g., length of training, lack of role models, financial difficulties, and a cultural gap between medicine and their graduate field).

Because these alumni trained decades ago, new research is needed to understand current SSHPH trainees’ experiences during MD-PhD training. As SSHPH students train in MD-PhD program environments that may be less familiar with their interests, characterizing contemporary experiences — including similarities versus differences relative to non-SSHPH peers — is critical. Therefore, our objective was to characterize the experiences of SSHPH MD-PhD trainees and compare them with non-SSHPH counterparts through an exploratory nationwide survey of current trainees.

## Results

### Participant characteristics.

Demographic data for 234 participants are available in Supplemental Table 1 (supplemental material available online with this article; https://doi.org/10.1172/jci.insight.199316DS1). The mean age of the cohort was 28.0 years (SD, 3.0; range, 21–41 years), with no significant difference between SSHPH and non-SSHPH students. More respondents identified as cisgender women than cisgender men (42.7%, *n* = 100 vs. 30.8%, *n* = 72, respectively). We combined respondents with diverse gender identities with those responses that were missing gender identity information to decrease identifiability (26.5% other gender or missing, *n* = 62). Racial diversity was low, with 54.3% of participants (*n* = 127) identifying as White and 11.1% (*n* = 26) as Asian. 8.1% of respondents (*n* = 19) were combined into a new category titled Underrepresented in MD-PhD training to decrease identifiability of participants. This category includes those who identify as American Indian or Alaska Native, Black or African American, Native Hawaiian or Pacific Islander, and mixed or multiple races. Data for 26.5% of the cohort was missing. Only 4.1% (*n* = 5) of non-SSHPH respondents identified as Hispanic/Latine, compared with 11.7% (*n* = 13) of SSHPH students (*P* = 0.03). Only a small proportion (12.4%, *n* = 29) identified as being from a low-income background, with no notable associations based on SSHPH status. Of note, our demographics were collected at the end of the survey, and there is a large percentage of missing responses.

Most participants (94.0%, *n* = 220) were in combined MD-PhD programs, with 76.1% (*n* = 178) in MSTPs. On average, they were midway through training (2.2 years of medical school and 2.5 years of graduate school completed). All regions of the US were represented, with 25.6% (*n* = 60) from the Midwest, 20.5% (*n* = 48) from the Southeast, 18.4% (*n* = 43) from the Northeast, and 12.0% (*n* = 28) from the West. Program names were not collected to decrease identifiability given the small numbers of SSHPH trainees at each school, but, based on an optional survey filled out by 74 participants interested in receiving notification of published results, students represented at least 27 unique institutions.

The most common SSHPH disciplines captured in our study were anthropology and epidemiology (each 27%, *n* = 30), along with fields such as health policy, science and technology studies, health behavior, philosophy/ethics, history, English/literature, and economics. Overall, 39.6% (*n* = 44) were in social sciences, 39.6% (*n* = 44) were in public health, and 8.1% (*n* = 9) were in humanities. Regardless of SSHPH status, most participants were interested in academic or academic medicine careers. SSHPH students were more likely than non-SSHPH participants to report interest in primary care (36.0% vs. 11.4%, *P* < 0.01) and less likely to report interest in industry (10.8% vs. 22.0%, *P* = 0.03). Nearly 70% of SSHPH participants and 58.5% of non-SSHPH participants responded to at least 1 free-text question.

### Sense of belonging.

Summary statistics of self-reported sense of belonging on a scale of 1–5 (not at all to extremely) are reported in Table 1 and Supplemental Table 2. SSHPH participants felt slightly less personally understood (3.5 vs. 3.8, *P* = 0.021), less welcomed based on their field of study (4.0 vs. 4.3, *P* < 0.01), and less respected based on their research interests than non-SSHPH participants (3.8 vs. 4.4 by students, *P* < 0.01 and 4.0 vs. 4.3 by program leadership, *P* = 0.013). Belonging within medical school was similar across groups, (3.2) but SSHPH participants reported higher belonging in graduate school (3.9 vs. 3.4, *P* < 0.01) compared with non-SSHPH peers. Overall sense of belonging in MD-PhD programs was lower for SSHPH students (3.7 vs. 4.0, *P* < 0.01).

Qualitative responses offered context for these patterns. Participants noted that their (a) research interests, (b) different training requirements, and (c) philosophical disciplinary differences — particularly the critical theoretical lens common in SSHPH fields — contributed to isolation and reduced belonging. Quotations exploring these themes are provided below.

One SSHPH student described comprehensively how these barriers contributed to a decreased sense of belonging: “I feel like we’re structurally marginalized from both MD students and PhD classmates as well as other MD/PhD students in basic sciences — as a result of different funding packages/financial concerns; different demands on our time and programmatic requirements/responsibilities; different job market prospects; different number of years of continuous training (fewer in each program, more overall); different intellectual orientations. These practical differences in the structure of our training mean we’re dealing with divergent material realities and personally has made it hard to have a sense of belonging with others.”

Another trainee described how they felt valued, yet gaps in understanding of their training path left room for additional belonging: “I also think that my MSTP leadership/the program values me, but I think they don’t really understand what I do which makes me feel lonely despite receiving positive support.”

Both SSHPH and non-SSHPH trainees referenced ideological disciplinary tensions. For example, one non-SSHPH participant was skeptical of SSHPH disciplines and viewed them as outside the MD-PhD mission (Supplemental Table 3): “Leave politics out of curriculum. We came here to learn biomedical science, not be indoctrinated in dubious theories on intersectionality from humanities departments or socialist ideology on equity.”

Other comments indicated that having greater numbers of SSHPH trainees in their programs increased their sense of belonging, compared with decreased belonging among transfer students (including transfers between institutions and/or transferring into MD-PhD programs mid-training), those training at multiple institutions, or those navigating major life events.

### Challenges and barriers.

Table 1 presents reported data on barriers and challenges experienced during training. Quantitative ratings of poor institutional support, personal responsibilities (e.g., care taking), first-generation status, personal illness (including mental health), and mistreatment/discrimination were similar between SSHPH and non-SSHPH participants (range of 1.8–2.6 overall, on a Likert scale of 1-5, from low to high, no significant differences). SSHPH students reported less stress related to the general challenges of MD-PhD training (e.g., academic demands of medical school; 2.5 vs. 3.0, *P* = 0.012) but more stress related to a lack of role models (2.7 vs. 2.1, *P* < 0.01) and uncertain job prospects (1.7 vs. 1.3, *P* = 0.042). Participants reporting “Other” challenges shared a diverse set of concerns, including anxiety about global politics, administrative requirements of medical and graduate school, and uncertain career goals.

Qualitatively, SSHPH participants described challenges and barriers related to (a) confusing application and recruitment policies from MD-PhD programs, (b) inexperienced research mentors for SSHPH MD-PhD students, (c) misaligned expectations from MD-PhD program leadership related to graduate milestones and measures of productivity, and (d) feeling like they fell into gaps between administrative systems, which required self-advocacy and extra work.

Students shared stories about being told that their institutions supported SSHPH training on websites or during interviews, yet they faced many roadblocks once arriving at the program, such as not finding any suitable SSHPH mentors prepared to work with MD-PhD students: “My program didn’t have a great sense of whether the broader institution would be capable of supporting my research goals. They accepted me to the mdphd program, but I realized pretty quickly… that I was going to have a very hard time finding research mentors here.”

Trainees described instances in which their program leadership tried to support them but struggled to do so: “In my experience, it was REALLY difficult to try to pursue a PhD in a “nontraditional” discipline. I think my MD/PhD admin folks tried their best to help me… but many of the PIs I reached out to had never even heard of an MD/PhD program… much less did they have the first idea of how to mentor an MD/PhD student.”

However, others described feeling like they did not having any advocates looking out for them: “I have often felt like an afterthought in the MD/PhD program and University… I have struggled with everything from being included on email list-servs, to registering for courses, to getting…a parking permit or ID card... I constantly feel that I have to advocate for myself - “explain” that I am a dual degree student in medicine and anthropology. It should not be MY responsibility to educate administrators about their own degree programs.”

### Funding and finances.

Table 1 describes the status of participants’ funding and finances. Overall, SSHPH participants reported more stress about funding than non-SSHPH students (2.5 vs. 2.1, *P* < 0.01). There were no significant differences in how SSHPH and non-SSHPH participants funded their medical school training, but SSHPH participants were significantly more likely than non-SSHPH participants to have service expected (e.g., teaching assistantships) for their base funding during graduate school (51.4% vs. 19.5%, *P* < 0.01). Guaranteed years of funding for medical school and graduate school did not differ between groups (3.8 and 4.0 years overall, respectively). About one-fifth of participants reported that funding affected their decision when choosing a graduate school department, with no differences based on SSHPH status. SSHPH participants were significantly more likely than non-SSHPH participants to report working additional jobs for pay (36.9% vs. 22.8%, *P* = 0.015), including jobs related to their professional goals (27.0% vs. 14.6%, *P* < 0.01). Levels of current undergraduate, medical school, and graduate school debt were not significantly different between groups.

Qualitatively, SSHPH students reported a variety of experiences with funding. Specifically, trainees described (a) a lack of funding parity between SSHPH and non-SSHPH disciplines, (b) differences in service requirements to secure base funding during graduate school, (c) lack of relevant grant opportunities for their scholarly work, and (d) longer training periods that increased funding stress. While many responses described differences in funding levels based on disciplinary groups, others described specific commitments from a few MD-PhD programs to pay students the same regardless of PhD discipline (e.g., supplementing SSHPH students for parity), although this sometimes still included differences in work requirements: “I specifically chose my institution because it funded its social science MD/PhDs at the same level as its science PhDs. I do think it’s frustrating though that I am required to TA and RA without additional pay while the science PhDs receiving the same funding don’t have to TA or can do so for more money.”

Participants commonly described a lack of institutional prioritization during graduate school years that were based at the university-level (i.e., beyond MD-PhD programs, mentors, or departments): “The university doesn’t care to provide a living wage to non-STEM students” and “the school of public health poorly funds graduate students and has a service expectation, making securing funding stressful.”

Participants also described challenges securing outside grants, writing “The way the F30 and F31 grant application process is structured, it’s difficult for us to apply. Often, what we’re working on doesn’t fit under a particular institute,” and “When I reached out to Pos [Project Officers] with my F30 specific aims for my proposed project, it was turned down in part because it was a global health project. Another student in my cohort lost points because their advisor does not have a history of NIH funding. This puts social sciences MD/PhD students at a huge disadvantage for receiving NIH funding.”

SSHPH and non-SSHPH students both reported concerns about broader structural equity, with concerns that those without outside support may fare worse economically. Trainees across disciplines also commonly advocated for higher stipends and adjustments for cost of living. A non-SSHPH trainee reported that “I am fortunate to not have debt only because I have a lot of parental financial support. I wouldn’t be able to afford this without them.”

### Recommendations for leadership.

Finally, participants provided recommendations for leadership to improve SSHPH training (Table 2). Trainees shared 5 domains of recommendations for MD-PhD directors. Each recommendation had several specific suggestions for how to enact these changes. For MD-PhD directors, trainees recommended (a) making intentional efforts to understand SSHPH research, training, and clinical paths, which involves seeking clarity about differences in SSHPH training, making active attempts to learn about diverse disciplines, and individualizing career support; (b) fostering relationships and mentorship with SSHPH faculty and leadership, including recruiting faculty role models, meeting with SSHPH faculty and departmental leaders, and supporting mentors unfamiliar with MD-PhD training; (c) creating a program that broadly benefits from the contributions of SSHPH disciplines through modeling appreciation of disciplinary diversity and sponsorship of inclusive program activities; (d) making the application process more streamlined by improving information available on websites, standardizing application processes, and recruiting more SSHPH students (with the condition that they would be supported once they join the program); and (e) advocating and working toward funding parity, including seeking out creative ways to fund students and looking to the models of programs that have made this a priority.

SSHPH trainees consistently emphasized the importance of program directors learning about their disciplines and valuing their contributions: “If a “non-traditional” (e.g., Public Health) PhD pathway is offered by the MD-PhD program, then program directors should take the time to learn about the structure of those programs just like they would know about Neuroscience or Microbiology.”

This also included understanding why their clinical interests may be distinct from traditional MD-PhD training paths: “When planning transition to residency, recognize that non-traditional PhDs may have research expertise that overlaps across multiple specialties; a general medicine track may be best suited for their career goals.”

Respondents described how students and university-wide scholarship could benefit from new relationships and collaborations across disciplines: “I’ve noticed that at my institution, our program director seems somewhat familiar with the biomedical research landscape at our institution and is extremely proactive in advocating for MD/PhD students... It seems that he is far less knowledgeable about what is going on in the social sciences departments, and I don’t even know if he’s ever spoken to my graduate program director. It would be nice if program directors could form these sorts of relationships beyond the biomedical sciences, not just for the sake of our students, but also for fostering greater interdisciplinarity at the university at large.”

Trainees described specific ways in which these contributions could be integrated into routine MD-PhD programming to the benefit of all students: “Please open space for our kinds of work in program-wide programming. Invite SSH students and faculty to speak to the entire MD-PhD [program]. Not to be flippant, but I have tried to take something away from many lectures about mice genes; and I deeply appreciate that my peers and program equally invite and take something away from lectures about the histories of medical racism, citation politics, or critical perspectives on epidemiology and disease outbreaks. These lectures are a hit!”

They also recommended better support for SSHPH mentorship: “Better support for mentors who may be unfamiliar with MD-PhD requirements, especially as it relates to funding mechanisms (e.g., basic science researchers are often more familiar with NIH funding mechanisms whereas social science researchers often look to other funding sources).”

Being fully transparent about the university’s ability to support SSHPH students — as well as decreasing barriers to applying to programs — was encouraged by students, who said, “Don’t accept students who are not doing doctoral work in programs you don’t support 100%” and “Do not require separate applications. Do not require MPHs, that is way too much school.”

Participants described the importance of advocating and prioritizing pay equity, with some providing examples of their programs enacting this: “Funding equity between the social sciences and other fields has been a priority of our program director since their tenure began. This is really key.”

The 3 recommendations for NIH included (a) valuing and promoting the training of SSHPH students (using MSTPs as a way to distribute structured support), which involves understanding and promoting the contributions of SSHPH trainees and creating metrics to reward programs with disciplinary diversity; (b) creating new funding and mentoring opportunities for SSHPH students, including adapting or creating additional grant opportunities for SSHPH students, providing SSHPH-relevant resources to help with grant writing, and supporting the development of new residency tracks; and (c) promoting funding parity through MSTP requirements.

Trainees often wanted the NIH to know why their work mattered and shared the following comments: “The history of American medicine has always acknowledged that politics, social factors, and the environment are all determinants to health. To not support… research in these areas, especially when they are orders of magnitude cheaper relative to wet lab research, is to do a disservice to the American public, many of whose health are more impacted by these issues than a ‘scientific’ question.”; “MD/PhDs in social science make very important contributions but require additional funding. There are so many amazing prospective students who would do great work, but not enough funded spots, so we’re missing out on a lot of much needed research as a result!”; and “Many of the problems of our healthcare system are beyond biomedical intervention — let us shed light on these problems and how to get past them!”

Participants described specific mechanisms by which these changes could be implemented: “[Provide] examples of successful F30/F31 applications in the social sciences” and “Need for additional funding opportunities that correlate to ‘traditional’ MD/PhD paths. Need for specialized research tracks in residency, especially!”

They described the MSTP designation as an important way to set funding expectations across programs: “Ensure that SSH PhDs are paid equally across all MSTP institutions. This should be a requirement for programs to maintain MSTP status if they want to train SSH students.”

Several trainees from both SSHPH and non-SSHPH disciplines reported concerns about mistreatment from mentors (Supplemental Table 4) and recommended that MD-PhD and NIH leaders more actively work to protect students from mistreatment, especially those who may be affected by other social inequities.

## Discussion

This exploratory study characterized the experiences of current MD-PhD trainees in SSHPH disciplines relative to peers in traditional biomedical science disciplines. Demographically, participating students were similar to non-SSHPH peers, except that more SSHPH respondents identified as Hispanic/Latine. SSHPH trainees in our sample were more interested in primary care and less interested in industry. While many training experiences were similar, SSHPH respondents showed small but directionally consistent differences in sense of belonging, specific challenges and barriers during training, and some funding and financial domains. Qualitative comments illustrated unique circumstances faced by some SSHPH trainees. Recommendations for MD-PhD programs and NIH leaders included better understanding of SSHPH training requirements and contributions as well as more active advocacy. Participants across disciplines emphasized the protecting students from mistreatment and improving funding.

### Sense of belonging and other challenges and barriers.

Our data show that participating SSHPH MD-PhD trainees report slightly lower sense of belonging in their MD-PhD programs but higher belonging in their graduate programs. Although overall respect for students’ research was relatively high, SSHPH trainees felt less respected by peers and leadership. Qualitative responses described dismissal of SSHPH topics and methods, limited understanding of disciplinary requirements, and isolation when being one of few SSHPH students. Importantly, there is immense heterogeneity between specific SSHPH fields of study, and even programs with multiple SSHPH trainees may overestimate similarity across disciplines (e.g., anthropology and epidemiology).

Students at institutions that promoted SSHPH speakers and/or built relationships with SSHPH faculty described feeling more welcomed and included. Adding such speakers to seminar series may be a simple way for MD-PhD students from all disciplines to be exposed to SSHPH scholarship. Building networks of SSHPH scholars interested in MD-PhD training — even across institutions — may promote community and shared support for SSHPH trainees.

### Funding and finances.

Findings related to funding were mixed, with some divergence between quantitative and qualitative results. Both SSHPH and non-SSHPH trainees described current stipend levels as insufficient. SSHPH students were far more likely to work additional jobs — over half reported doing so during graduate school, compared with less than 20% of non-SSHPH peers. SSHPH participants also reported higher stress related to funding and future job prospects. These findings suggest a more challenging financial landscape for SSHPH trainees. Addressing structural barriers may require creative solutions, such as using institutional overhead funds to supplement SSHPH trainees — a strategy that may become more difficult, yet increasingly important, in the context of NIH funding cuts.

### Trainee recommendations for MD-PhD and NIH leadership.

This study highlights the persistence of challenges (e.g., the need for increased mentorship and more institutional support) demonstrated in the 2017 SSHPH MD-PhD alumni study (9). Holmes et al.’s cohort received their degrees decades ago, most often outside of formal MD-PhD programs (9). While some improvements may reflect growing inclusion of SSHPH students in formal programs, our findings suggest additional targeted support is still needed.

A recent commentary advocating expansion of SSHPH MD-PhD training recommends 6 program-level action items: identifying key institutional advocates, integrating SSHPH into broader MD-PhD curricula, streamlining admissions, increasing undergraduate outreach, promoting training of additional SSHPH graduates, and engaging foundations, donors, and the private sector (8). For national efforts, they recommend increased funding and more residency tracks (8). Our survey provides empirical support that these interventions would be well-received by SSHPH students.

Several initiatives are underway to support SSHPH trainees. First, the American Physician Scientists Association (APSA) continues to prioritize SSHPH inclusion, maintaining an SSHPH program database and a dedicated representative on their leadership committee (10). Second, Making Physician-Scholar Trainees (MPHSTs, pronounced “Misfits) is a mentoring program that aims to help applicants navigate SSHPH MD-PhD admissions (11). Finally the Society for Humanities, Social Sciences, and Medicine — founded by SSHPH MD-PhD trainees in 2005 — remains a key event for presentations and community-building (12). The Society held its 10th Biennial Conference in April 2024 and inspired a new partner conference called the European Conference on Social Medicine, inaugurated in 2025 in Oslo, Norway. The Society most recently adjourned in San Francisco in April 2026 (13).

While all MD-PhD trainees face challenges, those from underrepresented backgrounds experience added barriers (14–16). Recruiting and retaining a diverse MD-PhD workforce is critical but increasingly difficult amidst restrictions on affirmative action and pipeline programs (17). Only 8% of our sample identified with an underrepresented racial identity and 12% of our cohort was from a low-income background, reflecting recent reports of declining economic diversity (18) and ongoing racial bias that persists in MD-PhD admissions (19). These findings underscore the need for inclusive recruitment and retention strategies beyond disciplinary diversity.

Importantly, major disruptions to NIH funding and science in the US may put constraints on the ability for programs and funders to implement some of these recommendations in the near future (20). Our hope is that some smaller-level changes (e.g., incorporation of SSHPH content into program curricula, fostering of relationships across disciplines) can be implemented quickly to ease challenges associated with SSHPH training while also recognizing that major changes (e.g., requirements for funding parity) may be easier to facilitate in future scientific policy environments.

### Limitations.

This exploratory study has several limitations. First, our survey includes substantial missing data due to many participants skipping questions or leaving the survey early. We had particularly high rates of missing data for personal demographic characteristics collected at the end of the survey, which we documented clearly throughout our results. Given the very high rates of missing data and assumptions that underlie survey weighting with high missingness, we opted not to conduct multivariate analyses. We are encouraged that 76% of our participants endorsed being part of an MSTP (which aligns with trends in previously published MD-PhD matriculant data) (21) and that we captured students from every region of the county, representing at least 27 unique institutions.

Second, our recruitment strategy could not guarantee outreach to all MD-PhD students in programs supporting SSHPH training, limiting generalizability. We were unable to calculate a response rate, as we lacked confirmation from all programs that distributed the survey and did not collect institutional data, and thus, we are also not able to assess for selection bias by institution type.

Finally, our survey may obscure heterogeneity between (a) training programs, (b) differences between social science and humanities students and those in public health (the latter of whom may be more integrated into their MD-PhD programs owing to closer alignment with biomedical paradigms) (22), and (c) the challenges that other “nontraditional” (e.g., biomedical engineering) students may face.

To our knowledge, this is the first study capturing the perspectives of current SSHPH MD-PhD trainees. Key strengths include nationwide participation from at least 27 unique institutions, input from over 110 SSHPH students, quantitative comparison with non-SSHPH peers, and practical recommendations for MD-PhD and NIH stakeholders. Future work is needed to understand how SSHPH MD-PhD students may be affected by current disruptions in US science and medicine, as these data are from 2023. Additionally, future surveys should use more rigorous sampling techniques to more accurately capture participant demographics.

### Conclusion.

Our exploratory nationwide survey found that while SSHPH and non-SSHPH MD-PhD trainees shared many experiences, SSHPH trainees reported challenges like decreased sense of belonging, difficulty identifying role models, and increased work requirements during graduate school. Participants also identified actionable recommendations for program leaders and the NIH, warranting future study of their effectiveness in supporting SSHPH physician-scholar trainees.

## Methods

### Study design.

A survey was administered to current US MD-PhD students from March to July 2023. Responses to quantitative and qualitative items were compared between self-identified SSHPH trainees and those in traditional biomedical disciplines.

### Survey development and measures.

The survey, developed with a social science survey expert, included 5 domains: sense of belonging, challenges/barriers, funding/finances, recommendations for leadership, and demographics (Supplemental Methods, Full Survey). Sense of belonging items were adapted from the Harvard-Panorama Student Perception Survey (23), the United Kingdom Student “Belongingness” Survey (24), and the Imperial College of London Sense of Belonging Scale (25). Four domains included both closed- and open-ended items; leadership recommendations were assessed only through open-ended questions.

### Recruitment.

Programs accepting SSHPH students were identified on the APSA (10) and the Association of American Medical College (26) websites (*N* = 53 programs, representing >3,900 enrolled MD-PhD trainees). Program contacts were emailed to share the survey via listservs, with alternative contacts sought when emails failed. Up to two reminders were sent between March and July 2023. Participating programs were not systematically tracked, as many schools did not confirm distribution, and program names were not collected in the survey to decrease identifiability given small numbers of SSHPH students; however, at least 27 unique institutions were represented based on a separate optional survey collecting email addresses for notification of published results. Both SSHPH and non-SSHPH students were intentionally recruited.

### Data collection.

Participants accessed the survey via Qualtrics and provided informed consent. No compensation was offered. At survey completion, participants could opt into a separate list to receive study results. Responses were collected March–July 2023.

### Comparison of quantitative and qualitative data.

Following a convergent parallel mixed-methods design, we analyzed quantitative and qualitative separately and then compared within each domain as a form of methodological triangulation (27, 28). Qualitative themes added nuance to quantitative results.

### Statistics.

We summarized data using counts and percentages for categorical variables and means and SD for numerical and Likert variables. Completion was defined as completing at least 1 domain of responses (e.g., sense of belonging) with subsequent nonresponses documented as missing. During analysis, we collapsed some demographic categories to reduce identifiability and provided means when ranges were provided (7–8 years to 7.5 years). Analyses were conducted in R v4.3.1 (29) and used Fisher’s exact tests (categorical), 2-tailed *t* tests (continuous), and Wilcoxon’s rank-sum tests (Likert), with SSHPH status (yes/no) as the independent variable. Statistical significance was set at *P* < 0.05.

### Qualitative analysis.

We structured a hybrid inductive-deductive approach (30) to identify themes in open-ended survey responses, focusing on SSHPH responses and comparative perspectives from non-SSHPH students. Investigator triangulation was used (27) to separately develop two versions of a draft codebook based on the survey domains and open-ended responses and then refined the combined codebook iteratively, with each domain having several subcategories that emerged during early data exploration (Supplemental Methods, Qualitative codebook). They jointly reviewed and discussed all statements together in real-time and coded responses to consensus using ATLAS.ti v 23.3.0 (31). Coded data were organized by domain, with themes described and illustrated using representative quotes, lightly edited for grammar and spelling.

### Study approval.

All enrolled participants provided written informed consent embedded in the Qualtrics survey. This study was designated exempt by the University of North Carolina at Chapel Hill’s IRB (no. 22-2438).

### Data availability.

The data underlying this study are maintained within the University of North Carolina secure data environment and are not publicly available due to privacy and institutional restrictions. Deidentified data may be made available to investigators upon reasonable written request to the corresponding author, contingent upon approval by University of North Carolina at Chapel Hill and execution of appropriate data use agreements and ethics approvals. Analytic code supporting the findings of this study is available from the corresponding author upon reasonable written request; it is not publicly posted as certain outputs contain small cell sizes that could compromise participant confidentiality.

## Author contributions

CS and ARK have over 10 years of qualitative research experience, which they used to structure a hybrid inductive-deductive approach (30) to identify themes in open-ended survey responses. CS and CRC used investigator triangulation to develop, review, and organize coded survey responses. All authors meet ICMJE guidelines for authorship. Conceptualization: CS, EKL, and ARK. Data curation: CS. Formal analysis: CS, CRC, and EKL. Investigation: CS, EKL, CRC, SMH, and ARK. Methodology: All authors. Project administration: CS. Resources: CS. Software: CS and EKL. Supervision: ARK. Validation: All authors. Visualization: All authors. Writing of the original draft: CS. Review and editing: All authors.

## Conflict of interest

The authors have declared that no conflict of interest exists.

## Funding support

This work is the result of NIH funding, in whole or in part, and is subject to the NIH Public Access Policy. Through acceptance of this federal funding, the NIH has been given a right to make the work publicly available in PubMed Central. No funds were available to directly support this project (e.g., participant incentives).

- Eunice Kennedy Shriver National Institute of Child Health and Human Development at the NIH (to CS; 1F30HD116454).
- National Institute on Aging at the NIH (to ARK; K01-AG084971).
- European Union through the European Research Council (to SMH; E101045424).
- Spanish Ministry of Science, Innovation and Universities (to SMH; CNS2023-133290).

## Supplementary Material

Supplemental data

## Figures and Tables

**Table 1:**
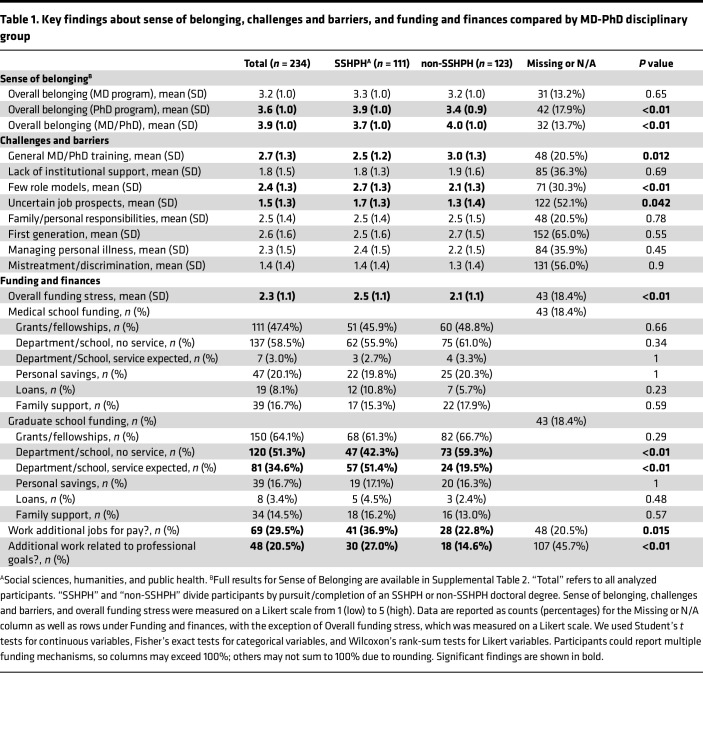
Key findings about sense of belonging, challenges and barriers, and funding and finances compared by MD-PhD disciplinary group

**Table 2:**
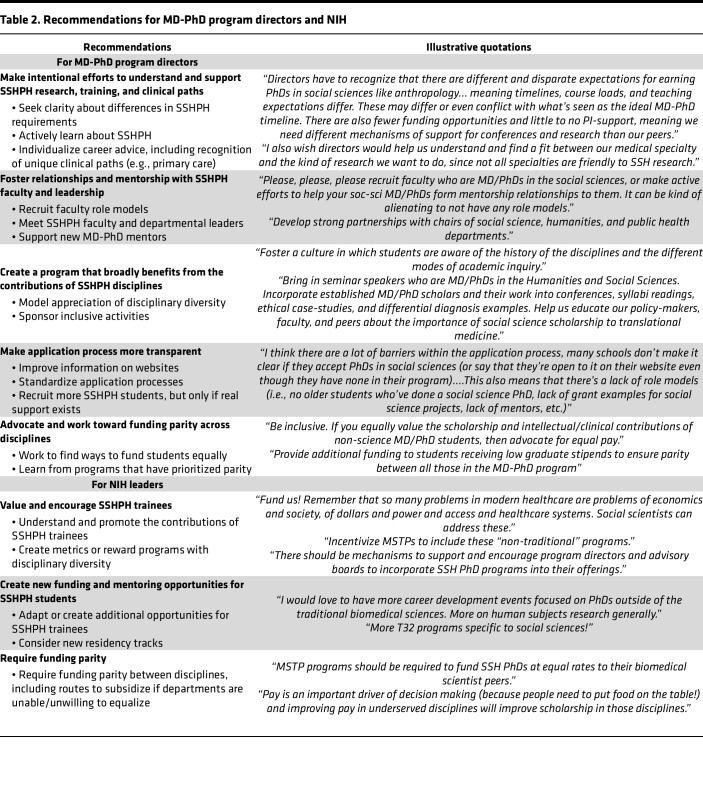
Recommendations for MD-PhD program directors and NIH

## Version 1 04/07/2026

In-Press Preview

## Version 2 05/22/2026

Electronic publication
