# Supplementary material for: Comparing MD-PhD trainee experiences by disciplinary background
Source: JCI Insight. 2026 Apr 7;11(10):e199316. doi: 10.1172/jci.insight.199316 (PMC13232710; doi:10.1172/jci.insight.199316)
Supplement: Supplemental data [file jciinsight-11-199316-s184.pdf]

Supplemental Results
Smith et al.

Supplemental Table 1: Participant characteristics. Social sciences, humanities, and public health ("SSHPH") and "non-SSHPH" columns split the total sample into two groups based on their pursuit/completion of an SSHPH or non-SSHPH graduate school degree, respectively. Numerical variables were summarized with a mean (standard deviation), categorical variables were summarized with a count (percentage). Missing data were summarized with a count (percentage) for any variable with greater than 0 missing responses. We used Student's t-tests for continuous variables and Fisher's exact tests for categorical variables to identify statistically significant differences between SSHPH and non-SSHPH participants. Columns may not sum to 100% due to rounding.

	**Total Sample (*N* = 234) n (%)**	**SSHPH[A] (*n* = 111) n (%)**	**non-SSHPH (*n* = 123) n (%)**	**p-value**
**Age** (mean, SD)	28.0 (3.0)	28.3 (3.0)	27.8 (3.0)	0.23
Missing[B]	60 (25.6%)	29 (26.1%)	31 (25.2%)	
**Gender**				0.7
Cisgender Woman	100 (42.7%)	48 (43.2%)	52 (42.3%)	
Cisgender Man	72 (30.8%)	31 (27.9%)	41 (33.3%)	
Trans, Nonbinary, Other Identity, or Missing[C]	62 (26.5%)	32 (28.8%)	30 (24.4%)	
**Race**				0.46
Asian	26 (11.1%)	8 (7.2%)	18 (14.6%)	
Underrepresented in MD-PhD Training and/or Mixed or Multiple Races[D]	19 (8.1%)	9 (8.1%)	10 (8.1%)	
White	127 (54.3%)	62 (55.9%)	65 (52.8%)	
Missing	62 (26.5%)	32 (28.8%)	30 (24.4%)	
**Hispanic or Latino/a/x**	**18 (7.7%)**	**13 (11.7%)**	**5 (4.1%)**	**0.026**
Missing	56 (23.9%)	28 (25.2%)	28 (22.8%)	
**Sexual Orientation**				0.06
Heterosexual	116 (49.6%)	47 (42.3%)	69 (56.1%)	
LGBQ+	48 (20.5%)	29 (26.1%)	19 (15.4%)	
Missing	70 (29.9%)	35 (31.5%)	35 (28.5%)	
**Raised in Low-income Household**	29 (12.4%)	11 (9.9%)	18 (14.6%)	0.42
Missing	59 (25.2%)	30 (27.0%)	29 (23.6%)	
**Total Years Expected**	8.2 (0.9)	8.4 (1.0)	8.1 (0.9)	0.055

Supplemental Results
Smith et al.

	**Total Sample (*N* = 234) n (%)**	**SSHPH[A] (*n* = 111) n (%)**	**non-SSHPH (*n* = 123) n (%)**	**p-value**
Missing	57 (24.4%)	28 (25.2%)	29 (23.6%)	
**Future Career Interests**				
Academia	154 (65.8%)	70 (63.1%)	84 (68.3%)	0.52
Academic Medicine	161 (68.8%)	75 (67.6%)	86 (69.9%)	1
Primary Care Medicine	**54 (23.1%)**	**40 (36.0%)**	**14 (11.4%)**	**<0.01**
Government	48 (20.5%)	24 (21.6%)	24 (19.5%)	0.62
Industry	**39 (16.7%)**	**12 (10.8%)**	**27 (22.0%)**	**0.03**
Private Practice	20 (8.5%)	8 (7.2%)	12 (9.8%)	0.64
Missing	54 (23.1%)	27 (24.3%)	27 (22.0%)	

[A]Social sciences, humanities, and public health
[B]Note that demographics were collected at the end of the survey and we had significant rates of non-response for these questions.
[C]Due to small numbers of respondents with diverse gender identities, these were combined with "Missing" to decrease identifiability.
[D]Due to small numbers of respondents with diverse racial identities, these were combined into a new category titled Underrepresented in MD-PhD training to decrease identifiability of participants. This category includes those who identify as American Indian or Alaska Native, Black or African American, Native Hawaiian or Pacific Islander, and Mixed or Multiple Races.

Supplemental Results
Smith et al.

Supplemental Table 2: Full Sense of Belonging Results. "Total Sample" refers to all participants included in our analysis. "SSHPH" and "non-SSHPH" columns split the total sample into two groups based on their pursuit/completion of an SSHPH or non-SSHPH graduate school degree, respectively. All variables were collected on a Likert scale from 1-5, with a mean (standard deviation) presented for each question. Values closer to 1 reflect a lack of belonging, while values closer to 5 indicate a strong sense of belonging. Missing data were summarized with a count (percentage). We used Wilcoxon rank sum tests to identify statistically significant differences between SSHPH and non-SSHPH participants.

	**Total (*n* = 234)**	**SSHPH[A] (*n* = 111)**	**non-SSHPH (*n* = 123)**	**Missing**	**p-value**
**How well do people understand you?**	**3.6 (1.0)**	**3.5 (1.0)**	**3.8 (1.0)**	31 (13.2%)	**0.021**
**How connected do you feel to leadership?**	3.4 (1.1)	3.3 (1.1)	3.6 (1.2)	29 (12.4%)	0.076
**How welcomed is your field of study?**	**4.2 (1.0)**	**4.0 (1.0)**	**4.3 (1.0)**	29 (12.4%)	**<0.01**
**How happy are you in your program?**	4.3 (0.8)	4.3 (0.8)	4.2 (0.9)	31 (13.2%)	0.47
**Do other students respect your research?**	**4.1 (0.9)**	**3.8 (0.9)**	**4.4 (0.7)**	34 (14.5%)	**<0.01**
**Does leadership respect your research?**	**4.1 (0.9)**	**4.0 (0.9)**	**4.3 (0.8)**	33 (14.1%)	**0.013**
**Do you matter to your program?**	3.7 (1.0)	3.5 (1.0)	3.8 (1.0)	35 (15.0%)	0.087
**Overall belonging (MD program)**	3.2 (1.0)	3.3 (1.0)	3.2 (1.0)	31 (13.2%)	0.65
**Overall belonging (PhD program)**	**3.6 (1.0)**	**3.9 (1.0)**	**3.4 (0.9)**	42 (17.9%)	**<0.01**
**Overall belonging (MD/PhD program)**	**3.9 (1.0)**	**3.7 (1.0)**	**4.0 (1.0)**	32 (13.7%)	**<0.01**

[A]Social sciences, humanities, and public health

Supplemental Results
Smith et al.

Supplemental Table 3. Social science, humanities, and public health trainees' challenges related to philosophical and ideological differences based on scholarly discipline

	**Illustrative quotations**
**Trainee concerns about scientism, positivism, political priorities, and commitment to specific career paths**	"Other MSTP students are often hostile to what they don't understand (i.e., historical or humanities work) and demonstrate a high degree of scientism."
	"From my personal experience, I have felt that program directors have seemed rather dismissive of our methodological approaches, especially when they don't fit into traditional notions of positivist science."
	"…I think perhaps the most difficult dimension of belonging comes from what seem to be different political sensibilities and the way those sensibilities shape my priorities and those of my peers or program. Coming from a more critical disciplinary formation, there are times where the pursuit of science feels inadequate and even conflictual with how I understand what I'm in this program to do versus what my peers express or what the program emphasizes."
	"Do not expect students not in a positivist field to think in a positivist orientation."
	"Though I am not formally in a SS/H/PH degree program, my research is no issues related to access and diseases of poverty, traditionally meaning limited publication in science, nature, cell. Thus, it's not valued in the way that more obvious topics are, and as a socially progressive person, I have not felt welcome in what it a relatively traditionalist program where around 50% of students go into industry or consulting at this point. Sad that those careers are valued, but the few of us interested in social justice medicine and research are frequently sharing stories of how we were told our research was, in my case "glorified family medicine" as if doing family medicine was a bad thing to start with?"

Supplemental Results
Smith et al.

Supplemental Results Table 4. Concerns about mistreatment in MD-PhD programs

**Recommendations from SSHPH and non-SSHPH students**	
**Protect from mistreatment and promote equity in MD-PhD training**	"STOP FUNDING ABUSIVE MENTORS PLEASE. I know several people who have R01 funding who have sexually, emotionally, and mentally abused friends and who have negatively impacted so many careers… Just please tell the NIH and MSTP programs to stop allowing abusive mentors to interview students, to have graduate students, etc. The fact that so many of my fellow students (and I) have had to switch PhD labs due to abuse (as defined in the legal system) is astounding."
	"Protect from abusive PIs and do not look the other way because they are well-funded."
	"Obtaining diversity in the workforce means supporting diversity in training. Those of us from diverse backgrounds (1st gen, poverty, POC) are going to have more challenges and a less smooth path to our degrees. We are more susceptible to abuse during training. My program director routinely uses language which makes it clear to students who struggle that he considers it their fault (ie. I should have been able to separate my emotions about being sexually assaulted by someone affiliated with my department from my work; I needed to get over the emotion of that experience to succeed). Ultimately, we lose diverse candidates along the path because the whole system was built for the 80% of students who come from families with doctors and faculty in their family already. I think the focus should be on positive outcomes for people with diverse backgrounds (and therefore research interests) more so than on recruiting these people in the first place. If we start to have positive experiences, we will be the recruitment."

Supplemental Methods

Smith et al.

Supplemental Methods A. Full Survey

After students provided consent and indicated they met eligibility criteria through initial screening questions:

We are interested in exploring the experiences of MD-PhD students, especially those in the Social Sciences, Humanities, and Public Health (SSHPH). To have a comparator group, we are also collecting responses from MD-PhD students in basic sciences and other disciplines that fall outside of SSHPH. We look forward to hearing about your experiences with MD-PhD training, including recommendations for how to strengthen it.

Are you an MD-PhD student in a combined MD-PhD program pursuing a PhD in the Social Sciences, Humanities, or Public Health

Yes/no

If you are not in a formally combined MD-PhD program, please explain how your training works: short answer

2 degrees within the same institution, no overarching program support
2 degrees at different institutions
Already completed MD or PhD before current program
Other - please provide a brief description of your training course

Only SSHPH: Discipline of study:

American Studies
Anthropology
Biostatistics
Communication
Economics
English
Environmental Sciences or Environmental Health
Epidemiology
Ethnic Studies
Geography
Health Behavior
Health Policy
History
Literature
Philosophy or ethics
Political Science or Public Policy
Psychology
Religion
Science and Technology Studies
Sociology
Other: write in

All: Is your program a Medical Scientist Training Program (MSTP)?

Yes/no

Part 1: Sense of Belonging

We are interested in understanding *sense of belonging* in MD-PhD programs among students.

Adapted from the Harvard-Panorama Student Perception Survey Scale (Gehlbach, 2015), UK student "belongingness" survey (Yorke, 2016), and Imperial College of London Center for Higher Education Research and Scholarship Sense of Belonging Scale (Imperial College of London, 2022)

When you respond to the questions below, think about yourself as a student in your MD-PhD program.

Matrix 1

1) How well do people in your MD-PhD program understand you as a person?
Not at all/slightly/somewhat/quite/extremely
2) How connected do you feel to the program staff and leadership in your MD-PhD program?
Not at all/slightly/somewhat/quite/extremely
3) How welcoming have you found your MD-PhD program to be toward your field of study?
Not at all/slightly/somewhat/quite/extremely
4) How happy are you with your choice to be a student in your MD-PhD program?
Not at all/slightly/somewhat/quite/extremely

Matrix 2

5) How much respect do students in your MD-PhD program have toward your research goals?
None/a little/some/quite a bit/tremendous amount
6) How much respect do staff and leadership in your MD-PhD program have toward your research goals?
None/a little/some/quite a bit/tremendous amount

Matrix 3

7) How much do you matter to others at your MD-PhD program?
Not at all/a bit/somewhat/quite a bit/tremendous amount
8) Rate your overall *sense of belonging* for
   a. Your MD-PhD program *Not at all/a bit/somewhat/quite a bit/tremendous amount*
   b. Your MD program *Not at all/a bit/somewhat/quite a bit/tremendous amount*
   c. Your PhD program *Not at all/a bit/somewhat/quite a bit/tremendous amount*
9) Please provide explanations for responses that you marked "none" or "a little bit." You may also include comments about other answers. (short answer – will use survey logic and flag)
   a. Explanations of "none"
   b. Explanations of "a little bit"
10) If any of these answers have changed over time, please share in what ways. (short answer)

In this section, we want to understand challenges and barriers that students may face in their MD-PhD training.

Part 2: Barriers before and during MD-PhD training

Supplemental Methods

Smith et al.

1) Please indicate if you have personally faced any of these barriers in your MD-PhD training (yes/no). Please select all that apply.
   First-generation/family and other personal responsibilities/funding and financial stressors/managing personal illness/lack of institutional support/general challenges of medical school and doctoral training curriculum/few role models/mistreatment or discrimination including racism/uncertain job prospects/lack of respect/other (describe)

2) *How significant are these barriers? (each one)*
   Rate options above
3) What top 3 barriers do you think MD-PhD programs should prioritize addressing for trainees in SSHPH? **OR** What barriers do you find most pressing to address for trainees during their graduate school years?
   Rate options above
4) Please provide any additional information on barriers you or others similar to you have faced. This can also include difficulties that took place before securing a spot in an MD-PhD program that impact your current training. (short answer)
5) Can you think of ways these can be addressed? If so, please describe. (short answer)

Part 3: Funding

We are interested in better understanding your experience with MD-PhD funding, including tuition and stipend support.

1) How would you rate your stress surrounding funding during your MD-PhD program? *None/a little/some/quite a bit/tremendous amount*
2) How are you funded in your MD-PhD program? Select all that apply
   a. Medical school (grants, fellowships, school funding, personal funding, other)
   b. Graduate school (grants, fellowships, school funding, personal funding, other)
3) What is your approximate annual stipend (guaranteed funding from your institution, NOT including extra jobs) ##
   a. Medical school ##
   b. Graduate school ##
4) How many years are you **guaranteed** funding from your institution?
   a. Med school
   b. Grad school
5) Does your stipend differ from other MD-PhD trainees in your program? Yes, it is higher/ Yes, it is lower / no/not sure If it differs, by how much (approximate)?
6) Does the number of years you are guaranteed funding differ from other MD-PhD trainees in your program?
   Yes, it is lower / no/not sure If it differs, by how much (approximate # of years)?

7) Did you know through which general mechanisms you would be funded before you joined your MD-PhD program? Yes/no
8) Did you know approximately how much you would be funded before you joined your MD-PhD program? Yes/no
   a. If yes: was this accurate and/or did this change?
9) Did funding impact your decision when you picked **an institution** for MD-PhD training ? Yes/no (explain for yes)
10) Did funding impact your decision when you picked your PhD **discipline**? Yes/no (explain for yes)

11) Do you work any additional jobs for pay? yes/no; please describe
    a. Are these related to your professional goals? Yes/no; please describe
12) Have you taken out student loans for your **MD-PhD training**? yes/no; please describe
13) Have you received parental or spousal support during your MD-PhD training? yes/no; please describe
14) Approximately how much student debt do you currently have? #
    a. Graduate training
    b. Undergraduate training
15) Please share any comments related to funding during your MD-PhD program. If your MD-PhD program funds students in different graduate disciplines equally, please share how they do that. Short answer

Part 4: Recommendations (all short answer)

1) What recommendations do you have for **MD-PhD program leaders** who train or are interested in training MD-PhDs in the social sciences, humanities, and public health?
2) What recommendations do you have for **university or graduate school leaders** who may be interacting with MD-PhD trainees like you?
3) What recommendations do you have for the **National Institutes of Health (NIH)** to better support physician-scientist trainees like you?

Part 5: Demographics

1) Age, #
2) Gender (cis woman, cis man, transwoman, transman, nonbinary, other (self-describe))
3) Sexuality (Heterosexual, homosexual, bisexual, other (self-describe))
4) Race/ethnicity (Hispanic/non + census groups)
5) Nationality, immigrant status
6) Low-income background
7) Medically underserved community
8) Total household income before taxes 2021
9) School name(s)
10) Progress in MD program (years):
11) Progress in PhD program (years):
12) Expected years (total) in PhD
13) Expected years (total) in MD-PhD program
14) Approximate number of other current MD-hD SSHPH trainees (total) in your institution
    a. If you are training at more than 1 institution, please list program A and program B
15) Future medical specialty (list); type of setting (academic, community, etc.)
16) Brief description of career goals (if comfortable sharing)

Is there anything else you would like the research team to know?

Thank you for your time and insight. We appreciate you sharing your thoughts. If you would like us to send you a copy of our findings once published, please share your email address here (link). These will be stored separately from your responses.

Supplemental Methods

Smith et al.

References:

Gehlbach, H. (2015). User Guide: Panorama Student Survey. Retrieved from https://www.panoramaed.com/panorama-student-survey

Imperial College of London. (2022). Sense of belonging scale. Retrieved from https://www.imperial.ac.uk/education-research/evaluation/what-can-i-evaluate/sense-of-belonging/tools-for-assessing-sense-of-belonging/sense-of-belonging-scale/

Yorke, M. (2016). The development and initial use of a survey of student 'belongingness,' engagement and self-confidence in UK higher education. *Assessment & Evaluation in Higher Education, 41*(1), 154-166.

Supplemental Methods

Smith et al.

Supplemental Methods B. Qualitative codebook

Responses can be double coded or split into multiple quotes. Default is coding only in the section that the response is included in, but if it includes information highly relevant to other sections, it can be included in both sections (e.g. funding).

A. **Sense of belonging**

A1. Transitions of program

A2. Academic fit
- A2a. Research interests – whether others think your work is important
- A2b. Training demands – understanding what your degree program requires

A3. Social fit
- A3a. Minoritized identity or mistreatment
- A3b. Perceived social support
- A3c. General sense of community – includes comparison across programs (MD vs PhD etc.)

A4. Ideological, cultural, or philosophical differences

A5. Other (COVID, multiple institutions)

B. **Challenges and barriers for SSHPH students** [funding challenges covered in C]

B1. SSHPH academic challenges
- B1a. Different training demands – more classes, single-author papers, etc.
- B1b. Leadership (MD-PhD or PI) doesn't understand MD-PhD path
- B1c. Lack of information about SSHPH application and/or pathway
- B1d. Available mentors and other resources [not funding]

B2. Mistreatment - sexism, racism, abusive mentors

B3. Personal Obstacles
- B3a. Mental and/or physical health
- B3b. Life events – COVID, death in family, having a child
- B3c. Difficulties of training – length, distance, material, etc.

C. **Funding and finances**

C1. SSHPH funding differences
- C1a. Payment set by departments
- C1b. Service requirements
- C1c. Mechanisms of parity
- C1d. Grant opportunities or lack thereof

C2. Non-SSHPH funding differences
- C2a. F30 or other bonuses
- C2b. Completing degree at different institutions
- C2c. Joining program late

C3. General thoughts about funding
- C3a. Difficulty living with stipend level
- C3b. Family contributions/generational wealth
- C3c. Unpredictable funding

D. Recommendations for MD-PhD and graduate school/program leaders

D1. Improving belonging
- D1a. Encouragement and receptivity to interests
- D1b. Improve social interactions and collegiality across disciplines
- D1c. Inclusive SSHPH activities

D2. Addressing challenges and barriers
- D2a. Mentorship (student or faculty) and advocacy for SSHPH
- D2b. Protection from mistreatment (bad mentors, marginalized backgrounds)
- D2c. Application and recruitment process for SSHPH
- D2d. Simplify and personalize training paths
- D2e. Increase equity in program
- D2f. Understand SSHPH better

D3. Financial sustainability
- D3a. Funding parity between students
- D3b. Strategies for funding

E. Recommendations for NIH

E1. Funding
- E1a. Increase base stipend
- E1b. Additional funding opportunities (e.g., for SSHPH, parental leave, etc.)
- E1c. Funding parity

E2. Career development and mentorship

E3. Protect against mistreatment

E4. Encouragement and receptivity to interests

F. Meta codes

F1. Not sure/discuss
F2. Great quotes
F3. Not coded
